# IL-10 regulates Th17 response to inhibit hepatobiliary injury caused by *Clonorchis sinensis* infection in C57BL/6J mice

**DOI:** 10.3389/fcimb.2022.994838

**Published:** 2022-10-13

**Authors:** Beibei Zhang, Jianling Wang, Man Liu, Qianqian Zhao, Guozhi Yu, Bo Zhang, Hui Hua, Jinyao Xu, Jing Li, Qian Yu, Stephane Koda, Yin-Hai Xu, Zhihua Jiang, Chao Yan, Kui-Yang Zheng

**Affiliations:** ^1^ Jiangsu Key Laboratory of Immunity and Metabolism, Xuzhou Laboratory of Infection and Immunity, Department of Pathogenic Biology and Immunology, Xuzhou Medical University, Xuzhou, China; ^2^ Department of Laboratory Medicine, The Affiliated Hospital of Xuzhou Medical University, Xuzhou, China; ^3^ Guangxi Key Laboratory for the Prevention and Control of Viral Hepatitis, Guangxi Zhuang Autonomous Region Center for Disease Control and Prevention, Nanning, China

**Keywords:** *Clonorchis sinensis*, biliary injury, interleukin-10, Th17 cells, infection

## Abstract

Clonorchiasis caused by *Clonorchis sinensis* is a mainly foodborne parasitic disease. It can lead to hepatobiliary duct inflammation, fibrosis, obstructive jaundice, liver cirrhosis, and even cholangiocarcinoma. Interleukin (IL)-10 is an immune-regulatory cytokine which plays an immunosuppressive role during infection. Our previous study found that IL-10 was increased in mice with *C. sinensis* infection. However, the role and mechanism of IL-10 playing in hepatobiliary injury induced by *C. sinensis* infection remain unknown. Herein, *Il10^+/+^* mice and *Il10^+/-^* C57BL/6J mice were infected with *C. sinensis*. It was found that IL-10 deficiency aggravated biliary hyperplasia and exacerbated periductal fibrosis induced by *C. sinensis* infection. Moreover, IL-10 deficiency increased CD4^+^T cells and CD8^+^T cells but not macrophages in the liver of mice with infection. There were no apparent differences in Th1 and Treg cells between *Il10^+/+^* and *Il10^+/-^* mice infected with *C. sinensis*. However, the proportion of Th17 cells in CD4^+^T cells in *Il10^+/-^* infected mice was significantly higher than that in *Il10^+/+^* infected mice. IL-10 deficiency also enhanced the increase of Th17 cells induced by ESPs stimulation *in vitro*. Taken together, our results suggest that IL-10 plays a protective role in hepatobiliary injury in C57BL/6J mice induced by *C. sinensis* infection *via* inhibiting Th17 cells, which could deepen our understanding of the immunopathology of clonorchiasis.

## Introduction

Clonorchiasisis is mainly prevalent in East Asia, which could lead to liver and biliary disorders like bile duct hyperplasia, obstructive jaundice, liver cirrhosis, and even cholangiocarcinoma (Qian et al., 2016). Humans and other mammals are infected by the consumption of raw freshwater fish containing *C. sinensis* metacercariae. Because of the long-term formation of customs and culture, changing inhabitants’ food habits is too difficult. Therefore, the prevalence of clonorchiasis in some endemic areas remains high, and it remains to be a great risk to public health and economic development (Na et al., 2020). Although large research was conducted to explore its pathogenesis mechanism, it remains largely unknown.

Adult worms dwell in the biliary system, which thus results in bile duct obstruction, mechanical injury by nutrition intake through the mucosa of bile ducts, and immunopathology injury (Kim et al., 2017b). The pathology is characterized by bile duct epithelial hyperplasia and periductal fibrosis (Na et al., 2020). In particular, immune responses are closely associated with the development of clonorchiasis. Our group and others have reported that T-helper cell subsets such as Th1, Th2, Th17, and Kupffer cells increased in the liver with *C. sinensis* infection (Zhang et al., 2017; Kim et al., 2017b; Kong et al., 2020; Wang et al., 2021). Type 1 immune response plays a role in clearing worms’ invasion during the acute stage. However, with chronic infection, Type 2-dominated immune response promotes the development of biliary fibrosis. Notably, the increase of Treg cells is also associated with biliary fibrosis (Yan et al., 2015). However, the detailed mechanism of the activation and differentiation of these sub-population immune cells in clonorchiasis is not fully clear.

Interleukin (IL)-10 is considered a cytokine with potent anti-inflammatory and immune-regulatory capabilities, which can influence antigen presentation, the differentiation of T cell subsets, and cytokine production (Boonpiyathad et al., 2019; Tang et al., 2020). It is produced by various cells such as Treg cells, macrophages, dendritic cells, and innate lymphoid cells (Akdis et al., 2016; Fang and Zhu., 2020; Koelink et al., 2020). IL-10 could modulate the function of CD4^+^ T cells directly. It can inhibit the secretion of IFN-γ and IL-2 by Th1 cells and downregulate IL-4 and IL-5 by Th2 cells (Boonpiyathad et al., 2019). The deficiency of IL-10 increases Th17 cells and accelerates the development of alphavirus encephalomyelitis (Kulcsar and Griffin, 2016). Besides, IL-10 suppresses the inflammatory effect of macrophages by negatively regulating inflammasome activation in a metabolic reprogramming manner (Ip et al., 2017). Our earlier studies reported that the expression of IL-10 increased in mice with *C. sinensis* infection (Kong et al., 2020). We also found that a ~34 kDa CsHscB purified from *C. sinensis* triggered IL-10 expression and therefore had the immune-regulatory ability (Yan et al., 2020). However, the exact role of IL-10 in the complex immune response in clonorchiasis is poorly understood.

In this current study, we evaluated the biliary injury in *C. sinensis*-infected C57BL/6J mice with IL-10 deficiency. The profiles of immune cells (CD8^+^T cells, CD4^+^T cell subsets, and macrophages) were investigated. We found that IL-10 deficiency exacerbated the hepatobiliary injury induced by *C. sinensis* infection and up-regulated Th17 cells. *In vitro*, IL-10 deficiency also enhanced the increase of Th17 cells stimulated by ESPs. Our study supports that IL-10 plays a protective role in clonorchiasis *via* modulating Th17 cells’ response in C57/BL6J mice. It might deepen our understanding of the link between *C. sinensis* infection and immune response in the host.

## Materials and methods

### Ethics statement

All experiments were strictly followed by the Guide for the Care and Use of Laboratory Animals of the National Institutes of Health. The experimental procedure followed the guidelines of the Committee for Animal Research of Xuzhou Medical University (201901w007).

### Mice and infection

C57BL/6J and *Il10^-/-^* (B6.129P2-*Il10^tm1Cgn^*/J) mice were obtained from Jackson Laboratories. All mice were maintained in a specific pathogen-free facility at the Xuzhou Medical University, which was housed with 22 °C-25 °C and 12 h day-night cycle. Water and food *ad libitum* were provided to feed animals. *Il10^+/+^* mice and *Il10^+/-^* mice used in experiments were strictly age and sex-matched. The genotypes and phenotypes of all mice we used were determined before the experiment and reconfirmed after sampling (**Supplementary Figure 1**).

A biliary injured mice model was established by infecting with metacercariae of *C. sinensis*. In brief, positive *pseudorasbora parva* were purchased from Guangxi Zhuang Autonomous Region, which were digested with pepsin overnight. The metacercariae were collected under an optical microscope. Each mouse was gavaged orally with 60 metacercariae and infected for six weeks. The normal groups were gavaged with saline.

### The preparation of the ESP

Eight-week-old guinea pigs were infected with 200 metacercariae of *C. sinensis.* After eight weeks, the livers were extracted for collecting adult worms. All worms were washed with phosphate-buffered saline (PBS) that contains 1% (v/v) penicillin/streptomycin several times and cultured in glass dishes using 1640 medium without phenol red. Cultured media was collected after 48 h and centrifugated at 1500 g/min for 30 min at 4 °C. Then, the supernatant was centrifugated at 3500 g/min for 30 min at 4 °C to remove worm eggs and cellular debris. At last, ESP was obtained by further centrifuging the supernatant at 12000 g/min for 45 min at 4 °C.

### Biochemical analysis

Serum was isolated by centrifuging the blood at 3500 rpm for 15 minutes. Alanine transaminase (ALT), aspartate aminotransferase (AST), and total bile acid (TBA) were detected in the Department of Laboratory Medicine, Affiliated Hospital of Xuzhou Medical University.

### Histology

Mice were anesthetized deeply and perfused with PBS. After mice were dissected, left livers with 1×1×1cm^3^ were sampled and fixed in 4% paraformaldehyde. After dehydration, clearing, and wax immersion, the paraffin sections were prepared for HE staining and Masson staining. The procedures of these two experiments followed the manufacturers’ instructions (Nanjing Jiancheng Bioengineering Institute, Nanjing, China). All images were captured under a microscope (Olympus, Tokyo, Japan). The histology activity index (HAI) was evaluated in a blinded fashion according to previously reported (Goodman, 2007). The image analysis of Masson staining was performed on Image-Pro Plus 6.0 software.

### RNA isolation and quantitative RT-PCR

To detect relative mRNA expression levels, TRIzol (TIANGEN, Beijing, China) was used to extract total RNA from the liver of each mouse. Nanodrop 2000 spectrophotometer was used to determine the quality and quantity of RNA. Then, 1 µg RNA was transcribed into cDNA followed by first-strand synthesis and reverse transcription with a First Strand cDNA Synthesis Kit (YEASEN, Shanghai, China). qPCR assays were performed as the following reaction procedure: 95°C for 5 min; 35 cycles of 95°C for 10 s, 60°C for 10 s and 72°C for 10 s; 60°C for 10 s, 70°C for 10 s. The relative gene expression was normalized with *Gapdh* and calculated using the 2^-ΔΔCt^ method. The primer sequences used to amplify the gene were respectively: *Gapdh* (Forward: 5’-AACGGATTTGGCCGTATTGG-3’; Reverse: 5’-CATTCTCGGCCTTGACTGTG-3’); *Acta2* (Forward: 5’-GTCCCTCTATGCCTCT GGAC-3’; Reverse: 5’-AAGGAATAGCCACGCTCAGT-3’); *Col1a* (Forward: 5’-TCCTGCGCCTAATGTCCACCGA-3’; Reverse: 5’-AAGCGACTGTTGC CTTCGCCTC-3’); *Rorc* (Forward: 5’-TGTGCCCACCACCTCACT-3’; Reverse: 5’-CCACCGTATTTGCCTTCA-3’); *Stat3* (Forward: 5’-GTTGGAGCAGCA TCTTCAGG-3’; Reverse: 5’-GCATGTCTCCTTGGCTCTTG-3’); *Il6* (Forward: 5’-GACTGATGCTGGTGACAACC-3’; Reverse: 5’-AGACAGGTCTGTTGGGA GTG-3’); *Il23* (Forward: 5’-GCAGCTCTCTCGGAATCTCT-3’; Reverse: 5’-TCCTTGTGGGTCACAACCAT-3’); *Tgfb* (Forward: 5’-TTGCTTCAGCTCCAC AGAGA-3’; Reverse: 5’-CAGAAGTTGGCATGGTAGCC-3’). Targeted mRNA expression levels were normalized to the housekeeping gene *Gapdh* and then fold changes were calculated by comparing with the expression of the controls.

### Preparation of spleen cells and culture

Eight-week-old *Il10^+/+^* mice and *Il10^+/-^* mice were immunized with 50 μg ESP subcutaneously. This was repeated two times with an interval of 14 days. The spleens were extracted 14 days after the last injection, and then ground with PBS containing 1% FBS (Gibco, Grand Island, USA). After centrifugation, ACK lysis buffer was used to remove the red blood cells. In detail, spleen cells were suspended with 1ml lysis buffer for 5 min on ice, and washed with 3 ml of PBS twice. These prepared cells were counted, and 2×10^6^ cells per well were seeded into a 24-well plate. Cells were cultured in 1640 complete medium with or without 50 μg/ml ESP. (Thermo, Waltham, USA), and maintained in a humidified atmosphere supplemented with 5% CO_2_ at 37°C. For cytokines detection, 25 ng/ml phorbol 12-myristate 13-acetate (PMA) and 1 μg/ml ionomycin (Sigma-Aldrich, ST. Louis, USA) were added to culture media. After 48 h, the supernatant was collected for cytokines detection, and cells were collected for analysis of the ratio of Th1, Th17 and Treg cells.

### Flow cytometry

The livers, spleens, and lymph nodes were harvested. All these issues were ground adequately, and filtered with a 70 μm filter membrane. To obtain the white blood cells from the liver, 40% and 70% percoll separation liquid (GE Healthcare, Pittsburgh, USA) were added and centrifugated at 2500 g for 20 min. Then, white blood cells were collected between these two layers of percoll separation liquid. For macrophage staining, cells were stained with 0.5 μl PB-anti-mouse F4/80 antibody (Pacific Blue, Shanghai, China) and 0.5 μl BV510 anti-mouse CD11b antibody (Biolegend, California, USA) for 30 min at room temperature. For Th1 and Th17 cells detection, 25 ng/ml phorbol 12-myristate 13-acetate (PMA) and 1 μg/ml ionomycin (Sigma-Aldrich, ST. Louis, USA) and monensin (Invitrogen, California, USA) were used to stimulate cells in culture medium for 5 h in a 5% CO_2_ chamber at 37°C in the dark. Then, cells were stained with APC-Cy7-anti-mouse CD3 antibody (Biosciences, Boston, USA), 0.5 μl Percp-cy5.5-anti-mouse CD4 antibody, and 0.5 μl APC-anti-mouse CD8 antibody (Biogens, Massachusetts, USA). Afterward, FIX&PERMkit (Invitrogen, California, USA) was used to fix and permeabilize cells. After fixation, cells were suspended with 100 μl permeabilization buffer and stained with 0.5 μl fluorescein isothiocyanate (FITC)-anti-mouse IFN-γ antibody and 0.5 μl PE-anti-mouse IL-17 antibody (Invitrogen, California, USA) for 15 min at room temperature. For detection of Foxp3, firstly, cells were stained with Percp-Cy5.5-anti-mouse CD4 antibody (Biosciences, Boston, USA) and 0.5 μl APC-anti-mouse CD25 antibody (eBioscience, California, USA) for 60 min at room temperature. After two washes with PBS, a Foxp3 Fixation/Permeabilization kit was used (eBioscience, California, USA). 800 μl of fixation was added to each tube and incubated at room temperature for 40 min. Then, 2 ml permeabilization buffer was added to each tube at 400 g for 5 min. Lastly, 0.8 μl PE-anti-mouse Foxp3 antibody (eBioscience, California, USA) was added and incubated at room temperature for 40 min in the dark. All data were obtained on the FACS Canto II system (BD Biosciences, New Jersey, USA) and analyzed by FlowJo software (Ashland, USA).

### Enzyme-linked immunosorbent assay (ELISA)

The concentration of IL-10 and IL-17 in the cell culture supernatant was detected by ELISA kits (Corning, New York, USA). Firstly, the 96-well plate (Corning, New York, USA) was coated with capture antibodies and incubated overnight at 4°C. After washing three times with washing buffer (PBS containing 0.05% Tween-20), each well was blocked with 200 µl ELISA/ELISPOT diluent at room temperature for 1 h. The plate was further washed 3 times with washing buffer. Samples were added and incubated at room temperature for 2 h. After washing five times, the detection antibodies were added and incubated at room temperature for 1 h. After another five times of washing, samples were incubated with avidin-HRP at room temperature for 30 min. Then, each well was washed seven times, and tetramethyl benzidine (TMB) was added and kept at room temperature for 30 min. 2 N HCl was used to stop the reaction. The optical density was read at 450 nm using a BioRad (Hercules, CA) ELISA reader. The concentrations of these cytokines were calculated according to the standard curve and normalized to the total protein concentration.

### Statistical analysis

Data are presented as mean ± SEM. Statistical analysis was conducted on SPSS 17.0. In detail, for comparisons between two groups, independent-sample *t*-tests were used. One-way ANOVA analysis (LSD test or Kruskal-Wallis H test) was performed for analyzing significant differences among multiple groups. Statistical significance was considered at a *P* value<0.05.

## Results

### IL-10 deficiency aggravates hepatobiliary injury caused by *C. sinensis* infection

**Figure 1:**
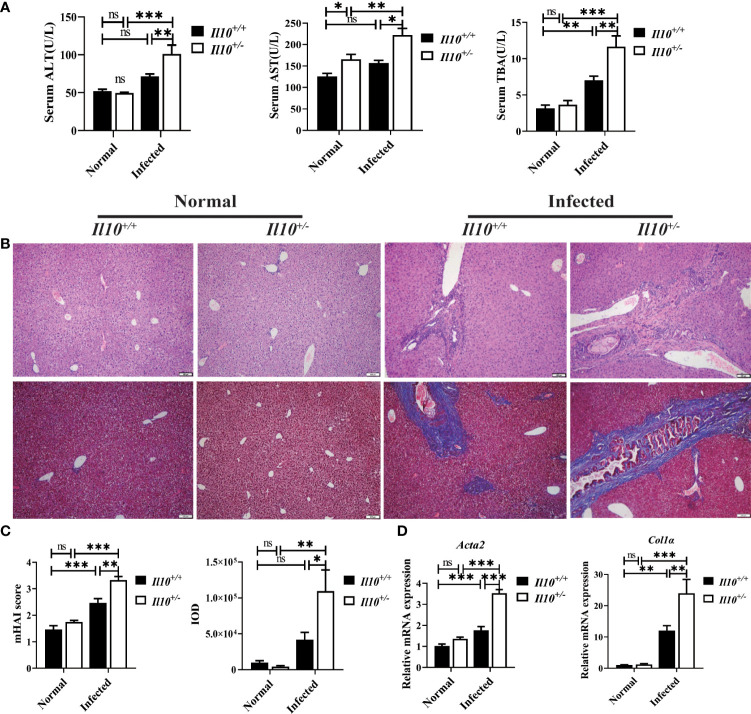
IL-10 deficiency aggravates hepatobiliary injury caused by *C sinensis* infection. **(A)** Serological levels of ALT, AST, and TBA. **(B)** Histopathological changes in the liver were observed by H&E staining, and fibrosis was examined by Masson’s trichrome staining. **(C)** The statistical analysis of mHAI score and fibrotic area in the liver. **(D)** The expression levels of *Acta2* and *Col1α* in the liver were detected by quantitative RT-PCR. Compared with indicated group, *P < 0.05, **P < 0.01, ***P < 0.001, ns, no significance.

To investigate the role of IL-10 in clonorchiasis, *Il10^+/-^* C57BL/6J mice were infected with 60 metacercariae of *C. sinensis*. As shown in **Figure 1**, *C. sinensis* infection slightly increased levels of serum ALT and AST in *Il10^+/+^* mice at 6 weeks post-infection (wpi). The TBA was higher in *Il10^+/+^* infected mice than in uninfected (*P*<0.01). In contrast, these indexes were much higher in *Il10^+/-^* infected mice than in *Il10^+/+^* infected mice (**Figure 1A**). Similarly, according to HE staining, it could be observed that the inflammatory cell infiltration and hyperplasia of biliary epithelium in *Il10^+/-^* infected mice were more evident than that in the *Il10^+/+^* infected mice. The HAI score was higher in *Il10^+/-^* infected mice (*P* < 0.01) (**Figures 1B, C**). Moreover, fibrosis formation could be observed around the bile duct in *Il10^+/+^* infected mice, and knockdown of IL-10 promoted the periductal fibrosis (*P* < 0.05) (**Figures 1B, C**). Additionally, the mRNA expression levels of two fibrosis-associated factors *Acta2* (*P* < 0.001) and *Col1a* (*P* < 0.01) (**Figure 1D**), were higher in *Il10^+/-^* infected mice than in *Il10^+/+^* infected mice. All these data suggested that IL-10 deficiency contributed to hepatobiliary injury in clonorchiasis.

### T cells but not macrophages are increased in clonorchiasis of *Il10^+/-^* mice

**Figure 2:**
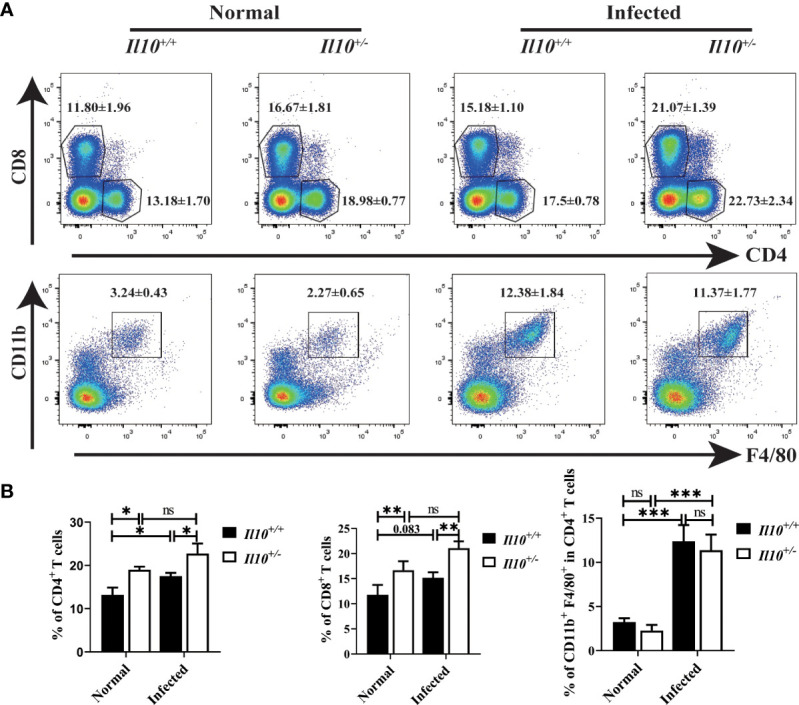
T cells but not macrophages are increased in clonorchiasis of *Il10^+/-^* mice. The mice were sacrificed at 6 wpi, and white blood cells were isolated from livers for CD4^+^T cells, CD8^+^T cells, and macrophages detection by flow cytometry. **(A)** The expressions of CD4, CD8, F4/80, and CD11b in white blood cells were analyzed by flow cytometry. **(B)** The ratio of CD4^+^T cells, CD8^+^T cells, and F4/80^+^CD11b^+^ macrophages was analyzed by FlowJo V10.8.1 software. Compared with indicated group, *P < 0.05, **P < 0.01, ***P < 0.001, ns, no significance.

To explore the role of IL-10 in regulating immune response, white blood cells in the liver were isolated by centrifugation through 40% and 70% percoll gradient. Compared with *Il10^+/+^* normal mice, CD4^+^ T cells increased significantly in *Il10^+/+^* infected mice (*P* < 0.05), and CD8^+^ T cells increased slightly (*P* = 0.083). Moreover, the proportion of CD4^+^ T cells (*P* < 0.05) and CD8^+^ T cells (*P* < 0.01) in *Il10^+/-^* infected mice were higher than those in *Il10^+/+^* infected mice (**Figures 2A, B**). It has been reported that hepatic macrophages played important roles in *C. sinensis* immune escape and liver tissue repair (Kim et al., 2017a). To clarify whether IL-10 contributed to the liver injury in clonorchiasis *via* modulating macrophages, CD11b^+^ F4/80^+^ macrophages in the liver were determined by Flow cytometry. It was shown that the proportion of CD11b^+^ F4/80^+^ macrophages was much higher in *Il10^+/+^* infected mice than in normal mice. But there was no obvious difference between *Il10^+/+^* and *Il10^+/-^* mice in infected groups. These results suggested that IL-10 deficiency promoted T cell response but not macrophages in the liver with clonorchiasis.

### IL-10 deficiency promotes Th17 cell differentiation in clonorchiasis of *Il10^+/-^* mice

**Figure 3:**
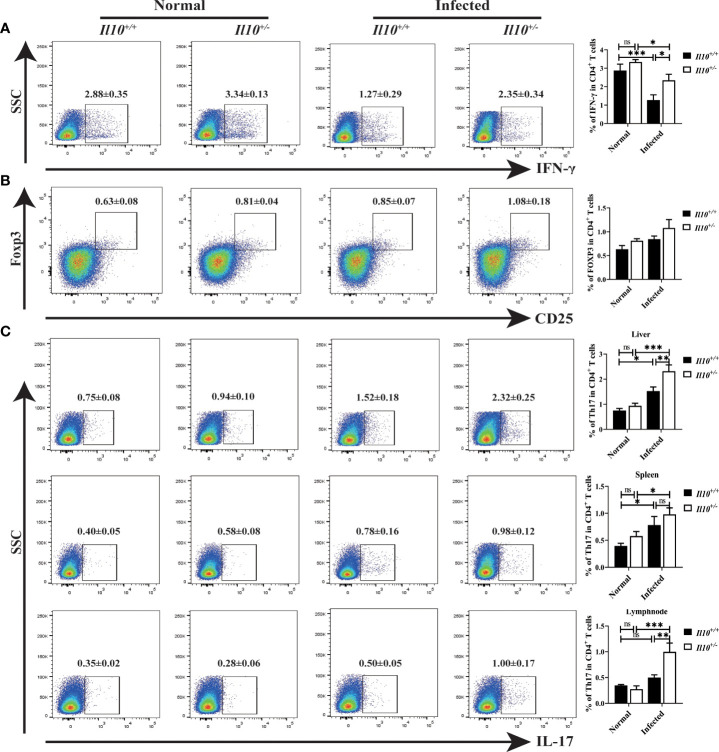
IL-10 deficiency promotes the ratio of Th17 in clonorchiasis of *Il10^+/-^* mice. **(A)** The percent of IFN-γ^+^ cells in CD4^+^ T cells in the liver was analyzed by flow cytometry (the left panel). The right panel shows the ratio of Th1 cells in CD4^+^ T cells. **(B)** The percent of CD25^+^ Foxp3^+^ cells in CD4^+^ T cells in the liver was analyzed by flow cytometry (the left panel). The right panel shows the ratio of CD25^+^Foxp3^+^Treg cells in CD4^+^ T cells. **(C)** The percent of IL-17^+^ cells in CD4^+^ T cells in the liver, spleen, and lymph node were analyzed by flow cytometry (the left panel). The right panel shows the ratio of Th17 cells in CD4^+^ T cells. Compared with indicated group, *P < 0.05, **P < 0.01, ***P < 0.001, ns, no significance.

**Figure 4:**
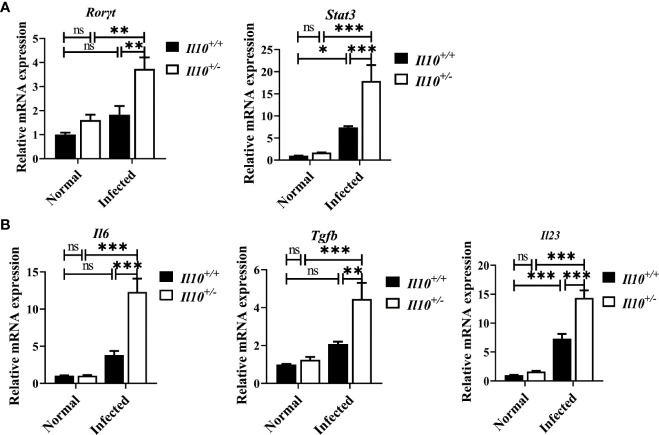
IL-10 deficiency promotes the transcriptional factors and associated cytokines of Th17 cells in clonorchiasis of *Il10^+/-^* mice. **(A)**The expression levels of *Rorγt* mRNA and *Stat3* mRNA. **(B)** The expression levels of *Il6*, *Tgfb*, and *Il23* mRNA in the liver were determined by quantitative RT-PCR. Compared with indicated group, *P < 0.05, **P < 0.01, ***P < 0.001, ns, no significance.

Next, we interrogated which CD4^+^T cell subset was regulated by IL-10 in infected mice. It was found that, compared with *Il10^+/+^* normal mice, the ratio of Th1 cells decreased significantly at 6 wpi (*P* < 0.001). Although IL-10 deficiency increased the ratio of Th1 cells in infected mice (**Figure 3A**), it was still lower than that in normal mice. Treg cell subset in the liver increased slightly in *Il10^+/+^* infected mice, and there were no obvious differences between *Il10^+/+^* and *Il10^+/-^* mice in infected groups (**Figure 3B**). Noticeably, IL-10 deficiency increased the ratio of Th17 cells in CD4^+^ T cells significantly in infected mice livers (*P* < 0.001) (**Figure 3C**). The ratio of Th17 cells was also determined in the spleen and lymph node, which also revealed a higher proportion of Th17 cells in *Il10^+/-^* infected mice than that in *Il10^+/+^* infected mice (**Figure 3C**). RT-qPCR was employed to detect the mRNA levels of two transcriptional factors of Th17 cells (STAT3 and RORγT), and three key cytokines associated with Th17 cells differentiation and generation (IL-23, IL-6, and TGF-β). As results shown in **Figure 4A**, the increase of *Rorγt* mRNA (*P* < 0.01) and *Stat3* mRNA (*P* < 0.001) caused by infection were enhanced by IL-10 deficiency. In addition, the mRNA levels of *Il23* (*P* < 0.001), *Il6* (*P* < 0.001), and *Tgfb* (*P* < 0.01) were markedly higher in *Il10^+/-^* infected mice than in *Il10^+/+^* infected mice (**Figure 4B**). Altogether, these results suggested that IL-10 deficiency promoted the recruitment and differentiation of Th17 cells but not Th1 and Treg cell subsets in clonorchiasis.

### The proportion of Th17 cells in CD4^+^T cells induced by ESPs stimulation is enhanced by IL-10 deficiency

**Figure 5:**
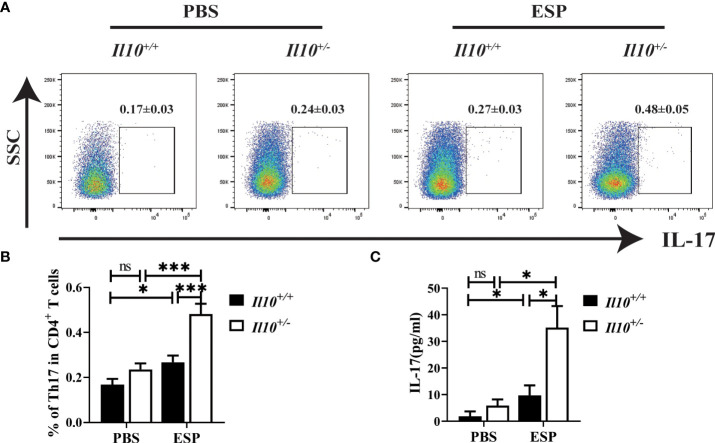
The increased proportion of Th17 cells in CD4^+^T cells induced by ESPs stimulation is enhanced with IL-10 deficiency. The *Il10^+/+^* mice and *Il10^+/-^* mice were sacrificed, and single-cell suspensions of splenocytes were prepared for ESP stimulation. **(A)** The expressions of IL-17 gated in CD4^+^ T cells are analyzed by flow cytometry. **(B)** The ratio of Th17 cells in CD4^+^ T cells. **(C)** The concentration of IL-17 in the supernatant was analyzed by ELISA. Compared with indicated group, *P < 0.05, ***P < 0.001, ns, no significance.

Secretory-excretory products (ESPs) released by *C. sinensis* flukes could spread to the biliary epithelium and liver, and therefore induce proliferation of biliary epithelium and trigger inflammation around the biliary tree (Na et al., 2020). To further verify the role of IL-10 in the regulation of Th17 cell responses in clonorchiasis, mice were immunized with ESPs, and splenocytes were collected to be stimulated with ESPs for 48 h *in vitro*. As shown in **Figures 5A, B**, ESPs significantly increased the proportion of Th17 cells in CD4^+^T cells (*P* < 0.05). Compared with splenocytes from *Il10^+/+^* mice stimulated with ESPs, a much higher proportion of Th17 cells was observed in *Il10^+/-^* group (*P* < 0.001). In addition, it also revealed that ESPs stimulation could promote more IL-17 secretion in the *Il10^+/-^* group than that in the *Il10^+/+^* group (**Figure 5C**, *P* < 0.05). These results indicated that IL-10 deficiency enhanced the Th17 response stimulated by ESPs, which confirmed the results *in vivo*.

## Discussion

Clonorchiasis is caused by adult worms dwelling in the biliary system, which could lead to biliary injury, obstructive jaundice, liver cirrhosis, and even cholangiocarcinoma. It has been reported that helper T cells and macrophages were associated with biliary injury in clonorchiasis (Zhang et al., 2017; Kim et al., 2017b; Kong et al., 2020; Wang et al., 2021). IL-10, a cytokine with potent immune-regulatory capacity, its exact role in clonorchiasis is poorly understood. In this study, we found that IL-10 deficiency could promote the biliary injury such as more severe inflammatory cells infiltration, hyperplasia of biliary epithelium, and periductal fibrosis *via* enhancing Th17 cells response, which supports a protective role of IL-10 in C57BL/6J mice infected with *C. sinensis*.

IL-10 is a regulatory cytokine that targets the immune system and controls the immune response to reduce tissue damage caused by various pathogen infections (Ouyang and O’Garra., 2019). Ablation of IL-10 resulted in minimal worm clearance and fatal tissue damage in *T. gondii*, malaria, and *Trypanosoma cruzi*-infected models (Saraiva et al., 2020). It supported to be a potential therapeutic target. It was reported that the level of IL-10 increased in mice infected with *C. sinensis* (Wang et al., 2021). However, its role in clonorchiasis remains to be studied. We and others have reported that the C57BL/6 mice strain is less susceptible than BABL/c and FVB mice, but also could result in obvious liver pathology (Uddin et al., 2012; Zhang et al., 2017). In addition, it has been reported that, compared with 4 weeks post-infection, the level of IL-10 decreased significantly in 8 weeks post-infection in C57BL/6 mice spleen. However, it showed no apparent differences in other mice strains, such as ICR, BALB/c, DDY, CAB/N and C3H/HeN (Uddin et al., 2012). Therefore, to further explore its role in hepatobiliary injury in this study, IL-10 deficient mice (B6.129P2-*Il10^tm1Cgn^*/J) were obtained from Jackson Laboratory. As is well acknowledged, IL-10-deficient mice can spontaneously develop a chronic inflammatory bowel disease associated with altered lymphocyte and myeloid profiles (Kuhn et al., 1993). But the severity of colitis is less severe on C57BL/6 genetic background than on other stains of mice (Berg et al., 1996). In addition, we found that mice with homozygotes of IL-10 mutant were in poor condition, showed bad mobility and loss of luster. According to our ELISA assay, the expression level of IL-10 in mice with heterozygous mutation (*Il10^+/-^* mice) in C57BL/6J genetic background decreased significantly. Thus, *Il10^+/-^* mice was considered to be used for *C. sinensis* infection. As results showed, compared with *Il10^+/+^* infected mice, ALT, AST and TBA were much higher in *Il10^+/-^* infected mice. Similarly, inflammatory cell infiltration, hyperplasia of biliary epithelium, and periductal fibrosis were also more severe in IL-10 deficient mice, indicating that IL-10 deficiency worsened the hepatobiliary injury, which also supported a protective role that IL-10 played in C57BL/6J mice with *C. sinensis* infection.

In response to diverse stimuli, several cell types secrete IL-10, such as CD4^+^ T cells, CD8^+^ T cells, macrophages, neutrophils, and eosinophils. IL-10 in turn results in an anti-inflammatory activity by targeting multiple cell types (Wang et al., 2019; Saraiva et al., 2020). CD4^+^ T cells, a major subset of T cells, play a vital role in fighting against parasitic infections *via* regulating host immune response (Bouchery et al., 2014). Meanwhile, CD8^+^ T cell response, including antigen presentation and the differentiation of effector T cells, is also an essential aspect of host resistance to parasite invasion (Tsitsiklis et al., 2019; Acosta Rodriguez et al., 2019). In our present study, we found that CD4^+^ T cells were increased significantly in *Il10^+/+^* C57BL/6J mice with *C. sinensis* infection for 6 weeks while CD8^+^ T cells were increased slightly. Wang and her colleagues have reported that CD4^+^ T cells were decreased while CD8^+^ T cells were increased in BALB/c strain mice with *C. sinensis* infection (Wang et al., 2021). The susceptibility to *C. sinensis* infection of C57BL/6 or BALB/c genetic backgrounds was reported to be different. In contrast to BALB/c infected mice, the recovery of the worm in C57BL/6 mice was less, and the development of worms was also dampened, which may contribute to a limited lesion of the tissue (Uddin et al., 2012; Zhang et al., 2017). This might result in discrepant responses of these two major T cell subsets. In this study, we found that IL-10 deficiency accelerated the increase of CD4^+^ T cells and CD8^+^ T cells. Apart from T cells, macrophages are essential components in promoting inflammatory responses, regulating tissue repair, and maintaining organ function. Consistent with others’ work (Kim et al., 2017a), our result also showed that CD11b^+^ F4/80^+^ macrophages were markedly induced by *C. sinensis* infection. However, IL-10 deficiency did not influence macrophages’ response. These data suggest that IL-10 regulates T cell response but not macrophages in a resistant mouse strain infected with *C. sinensis* infection.

CD4^+^ T cells play various roles in the adaptive immune system and are classified into four major subsets, including Th1, Th2, Th17, and Treg cells (Miggelbrink et al., 2021). Parasite antigens could evoke a dominant Th1 response during the acute stage of schistosomiasis, which then initiates the granulomatous inflammation (Zheng et al., 2020). In the chronic stage, there is a Th response transition from Th1 to Th2 response. Previously, it was reported that Th1 cells showed no changes at 4 wpi compared with normal C57BL/6 mice (Zhang et al., 2017). And in the present study, the ratio of Th1 cells was lower in *Il10^+/+^* infected mice at 6 wpi than in normal mice. All this seems to indicate that the Th1 response could not explain the liver pathology at this infection stage in the C57BL/6 resistant mice strain. Treg, an immunosuppressive subset of CD4^+^ T cells, is a potential therapeutic approach to autoimmune diseases, such as systemic lupus erythematosus, multiple sclerosis, and inflammatory bowel disease (Raffin et al., 2020). Recently, Xu *et al.* reported that the adoptive transfer of Treg could reverse the pro-inflammatory phenotypes and behavioral abnormalities induced by the soluble tachyzoite antigen from *Toxoplasma gondii* (Xu et al., 2021). Compared with more susceptibility FVB and BALB/c infected-mice models, the pathological changes are relatively mild in C57BL/6 mice (Zhang et al., 2017). The ratio of Treg also increases slightly in C57BL/6 infected mice at 6 wpi, which is consistent with our previous study (Zhang et al., 2017). Furthermore, there were no obvious differences in Th1 and Treg subsets between *Il10^+/+^* and *Il10^+/-^* mice in infected groups. All these data suggest that Th1 and Treg make a limited contribution to hepatic pathology in C57BL/6 infected mice, and are IL-10 independent.

Th17 cells, a vital cell subset of CD4^+^ T cells, increased significantly in infected mice livers. As is well known, Th17 cells exemplify immune adaptation. They are pathogenic mediators that contribute to inflammation and the pathogenesis of autoimmune diseases (Wu and Wan., 2020). In helminth-induced liver immunopathology, *Echinococcus granulosus* protoscoleces ESPs promote the differentiation of Th17 cells and the level of IL-17 (Pan et al., 2017). It has been clarified that Th17 and its associated cytokine IL-17 aggravate hepatic schistosomiasis (Zheng et al., 2020). We found that the ratio of Th17 cells kept stable at 4 wpi in C57BL/6 mice with *C. sinensis* infection (Zhang et al., 2017). In this study, with an infection lasting up to 6 weeks, Th17 showed a significant elevation, which indicated a contributing role of Th17 played in hepatobiliary injury caused by *C. sinensis*. Mechanistic studies indicate that the upregulation of IL-10 is negatively correlated with IL-17 through downregulating RORγt, which is the lineage-specific transcription factor of Th17 cells (Cerboni et al., 2021). Whether IL-10 modulates hepatobiliary injury caused by *C. sinensis via* acting on Th17 cell differentiation or not, is currently unclear and needs further investigation.

Herein, we found that the ratio of Th17 cells in *Il10^+/-^* infected mice livers was higher than that in the *Il10^+/+^* infected mice. This is also confirmed in the spleen and lymph node. Similar to the result obtained *in vivo*, IL-10 deficiency also strengthened the differentiation of Th17 stimulated by secretory-excretory products (ESPs) *in vitro*. The local cytokine microenvironment has been shown to determine T cell differentiation (Cerboni et al., 2021). It was well-known that TGF-β and IL-6 played critical roles in driving the differentiation of naïve Th cells into Th17 cells (Heink et al., 2017; Zhang, 2018). In detail, TGF-β and IL-6 could promote the expression of *Rorc* mRNA *via* activating STAT3, which further encodes RORγt transcription. IL-23 is a dispensable cytokine for Th17 cell differentiation, but vital for stabilization and proliferation (Heink et al., 2017; Zhang, 2018). IL-23/IL-23R signaling activates STAT3 pathways, and thus promotes disease pathogenesis. In our present study, we found that IL-10 deficiency enhanced the expression of key transcription factors (RORγt and STAT3), and cytokines (TGF-β, IL-6, and IL-23). All these data confirmed that IL-10 deficiency promoted the differentiation of Th17 cells in *C. sinensis*-infected C57BL/6J mice. And whether IL-10 could also regulate hepatobiliary injury *via* Th17 response in more susceptible *C. sinensis*-infected mice models or not, is worthy of being verified.

In summary, we identified IL-10 protected mice from hepatobiliary injury induced by *C. sinensis* infection. The protective role is based on inhibiting Th17 cell response. Promoting IL-10 production might be a potential strategy to treat clonorchiasis.

## Data availability statement

The original contributions presented in the study are included in the article/**Supplementary Material**. Further inquiries can be directed to the corresponding authors.

## Ethics statement

All experiments were strictly followed by the Guide for the Care and Use of Laboratory Animals of the National Institutes of Health. The experimental procedure was following the guidelines of the Committee for Animal Research of Xuzhou Medical University (201901w007).

## Author contributions

K-YZ, CY, and BBZ designed the experiments. BBZ, JW, ML, QZ, GY, BZ, HH, JX, JL, SK and Y-HX performed the experiments. ZJ collected the *C. sinensis* positive *pseudorasbora parva*. BBZ, JW, and QY contributed to the data analysis. BBZ and JW wrote the paper. K-YZ and CY reviewed the final version of the manuscript and supervised the project. All authors read and approved the final version of the manuscript.

## Funding

This study was supported by the National Natural Science Foundation of China (Grant Nos: 82172297 to K-YZ), Natural Science Foundation of Jiangsu Province of China (Grant No. BK20211346 to CY and BK20201011 to BBZ), Natural Science Research of Jiangsu Higher Education Institutions of China (Grant No. 20KJB310011 to BBZ), Jiangsu Postdoctoral Science Foundation (No. RC7062005 to BBZ), the starting grants for young scientist of Xuzhou Medical University (No. D2019040 to BBZ), Priority Academic Program Development of Jiangsu Higher Education Institutions of China (K-YZ) and Graduate research project of Jiangsu Province (Grant No. KYCX21-2468 to JW). The funders had no role in study design, data collection, and analysis, decision to publish, or preparation of the manuscript.

## Acknowledgments

We thank the Chinese Center for Disease Control and Prevention for providing us with *pseudorasbora parva* infected with *C. sinensis* metacercariae, and the Department of Laboratory Medicine, Affiliated Hospital of Xuzhou Medical University for supplying liver function test.

## Conflict of interest

The authors declare that the research was conducted in the absence of any commercial or financial relationships that could be construed as a potential conflict of interest.

## Publisher’s note

All claims expressed in this article are solely those of the authors and do not necessarily represent those of their affiliated organizations, or those of the publisher, the editors and the reviewers. Any product that may be evaluated in this article, or claim that may be made by its manufacturer, is not guaranteed or endorsed by the publisher.
