# Supplementary material for: IL-10 regulates Th17 response to inhibit hepatobiliary injury caused by Clonorchis sinensis infection in C57BL/6J mice
Source: Front Cell Infect Microbiol. 2022 Oct 13;12:994838. doi: 10.3389/fcimb.2022.994838 (PMC9606589; doi:10.3389/fcimb.2022.994838)
Supplement: Supplementary file 1 [file DataSheet_1.docx]

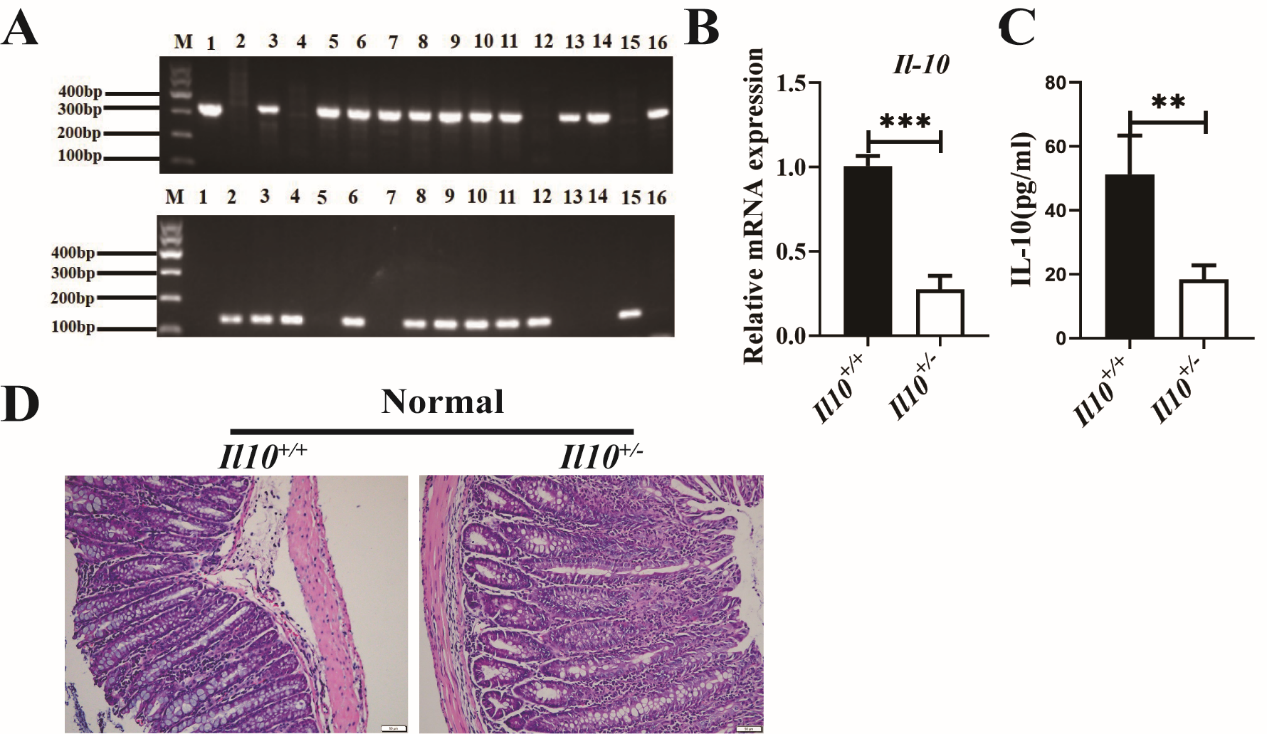


**Supplementary Figure 1.** **The genotype validation of *Il10^+/-^* mice. (A)** The genotype of *Il10^+/-^* mice was detected by PCR. The amplification band of *Il10* knockout is 312 bp, and the wild type is 137 bp. The first lane named M is DNA markers. The lanes named 2, 4, 12, 15 indicate wild-type genotype (*Il10^+/+^*), 3, 6, 8, 9, 10, and 11 indicate heterozygous mutant genotype (*Il10^+/-^*), 1, 5, 7, 13, 14, and 16 indicate homozygous mutant phenotype (*Il10^-/-^*). **(B)** The expression level of *Il-10* mRNA decreases in the *Il10^+/-^* mouse liver. **(C)** Spleen cells were cultured in 1640 complete medium for 48 h. Compared with *Il10^+/+^* mice, The concentration of IL-10 in the supernatant of *Il10^+/-^* spleen cells is significantly lower than *Il10^+/+^*. **(D)** H&E staining shows no obvious histopathological changes in the colon of *Il10^+/-^* mice. Compared with indicated group, * *P* <0.05, ** *P* <0.01.
